# Platinum Group Metal (PGM) free multi metallic nanomaterial: a potential electrocatalyst for Ethanol Oxidation

**DOI:** 10.55730/1300-0527.3709

**Published:** 2024-10-14

**Authors:** Susmita SINGH, Prodipta PAL, Soumik ROY, Shalini BASAK, Prantica SAHA, Anushna DUTTA, Sinthia SAHA, Mainak BOSE

**Affiliations:** Department of Chemistry, Amity Institute of Applied Sciences, Amity University, Kolkata, India

**Keywords:** Electrocatalysts, ethanol oxidation, low temperature fuel cell, nonnoble metal

## Abstract

Comprehensive studies of the ethanol oxidation reaction (EOR) have shown high interest in fuel cell technologies. As anode catalysts, introducing platinum group metal (PGM) free catalyst is promising for higher catalytic activity towards the EOR, as these are cost-effective, pollution-tolerant, and suitable for sustainable energy conversion. In this investigation, multi walled carbon nanotube (MWCNT) supported PGM-free electrocatalysts are synthesized by the impregnation reduction method. The atomic structure, composition, and morphology of nanoalloy catalysts are discovered through X-ray diffraction (XRD), Raman spectroscopy and fourier-transform infrared (FTIR) spectroscopy techniques. Electrochemical behaviours have been analysed by cyclic voltammetry (CV), linear sweep voltammetry (LSV), Chronoamperometry (CA), and electrochemical impedance spectroscopy (EIS), which reveal the oxidation kinetics of ethanol in an alkaline medium on the surface of the catalyst. The structure-activity relationship is a portrait of all the physical and electrochemical analyses that assists in exploring the active site of the surface, which facilitates electrooxidation activity. The C/Fe_50_Co_50_ catalyst exhibits higher catalytic efficiency and promotes CO removal through a bifunctional mechanism and electronic effect.

## 1. Introduction

With the perturbation of the climate crisis and rising demand for renewable green energy, effective energy conversion and sustainable energy sources have been explored worldwide. Fuel cells are regarded as among the most worthwhile technological alternatives as they provide a higher energy density, are environmentally benign, and have higher fuel utilization efficiencies than conventional combustion engines. Among all the Fuel cells, direct ethanol fuel cells (DEFCs) are one of the most promising fuel cells at room temperature, as ethanol has a higher specific energy (8.0 kWh/kg) than methanol and generates clean energy for power plants and transport systems [1–3]. The importance of ethanol oxidation is that it produces more energy compared to methanol as it involves a 12-electron process, while oxidation of methanol is a 6-electron process. Also, oxidation of ethanol in alkaline media has faster kinetics than in acidic media, due to an increase in the pH of electrolyte, which causes the potential to shift negatively. This shift affects the structure of the electric double layer and the distribution of the electric field at the interface between the electrolyte and electrode, thereby boosting the electrocatalytic activity. Conventionally, as electrocatalysts, Platinum group metals (PGMs) are used for hydrogen oxidation reactions (HOR) to achieve the needed current densities for fuel cells. However, unreasonable prices, susceptibility to CO poisoning, and insufficient presence of those metals hamstring the higher range commercialization [4–6]. As an alternative to PGM-free catalysts, discovering inexpensive, abundant materials have been the main aim globally [7]. Among the several studies of low costed transition metals grabbed a lot of interest. Fe is the one of transition metals, abundantly available on earth, cost-effective, exhibits a number of oxidation state and has the ability to interact with other elements by employing its valence electrons, plays promising role as a suitable catalyst for small organic molecules in alkaline environments. Also, it has a large surface area and promising adsorption capacity [8]. Despite these advantages, further research is needed to optimize iron-based catalysts for efficient electrooxidation of alcohols to increase the catalytic activity towards ethanol oxidation as suggested by different report [9]. To enhance iron’s catalytic performance, researchers have explored the synergistic effects of combining Fe with a second transition metal such as Co, Ni, which can promote the catalytic activity by lowering the CO poisoning on its surface and enhancing surface activity several times while making it economical. Among other metals, Cobalt is the oxophilic transition metal suitable for providing –OH species at lower overpotential. J Datta et al. [10] reported that addition of Co, can potentially serve as an improved catalyst for the electrooxidation of alcohol by intermediate adsorption and promote the CO_2_ removal, which is described in following equation -


(1) $$\text{M}({\text{CH}}_{3}\text{CO})+\text{O}-\text{Co}-\text{O}-\text{Co}\to {\text{CH}}_{3}\text{O}-\text{Co}-\text{O}-\text{Co}+\text{M}-\text{CO}$$

(2) $${\text{CH}}_{3}\text{O}-\text{Co}-\text{O}-\text{Co}+\text{M}-\text{OH}\to {\text{CH}}_{3}\text{O}-\text{Co}-\text{O}-\text{Co}-\text{OH}+\text{M}$$

(3) $${\text{CH}}_{3}\text{O}-\text{Co}-\text{O}-\text{Co}-\text{OH}-4{\text{e}}^{-}\to {\text{CO}}_{2}+\text{Co}-\text{O}-\text{Co}+4{\text{H}}^{+}$$

The novelty of developing iron-based catalysts is to enhance the catalytic efficiency at a lower overpotential, which is sustainable for the environment and influential for the feasible application of commercial fuel cell technologies. Therefore, the carbon-supported FeCo catalyst is a novel electrocatalyst for ethanol oxidation reaction in DEFC due to its enhanced catalytic performance, stability, and efficiency. Its innovative combination of metal alloy and carbon support offers significant improvements over traditional catalytic systems, making it a promising candidate for advancing sustainable and cost-effective DEFC technologies. In this research, MWCNT supported monometallic and bimetallic FeCo synthesized in 50:50, 84:16, and 91:9 ratios and unsupported FeCo using the impregnation-reduction method, aiming to create a cost-effective catalyst with high-performance capabilities for ethanol electrooxidation. This impregnation reduction method offers several advantages, including cost-effectiveness and the ability to tune the size and shape of the C/FeCo catalyst. For physical characterization, several techniques such as XRD, FESEM, FTIR spectroscopy, and Raman spectroscopy were adopted. Employed CV, LSV, CA and EIS to evaluate its electrochemical performance.

## 2. Materials and methods

### 2.1. Materials

All chemicals were utilized in this study exactly as supplied. The utilized chemicals were Fe(NO_3_)_3_.9H_2_O (SRL), Co(NO_3_)_2_.6H_2_O (SRL), NaBH_4_ (Merck), Ethanol (Merck), Multi Walled Carbon Nanotubes (MWCNTs) (Sisco Research Laboratories Pvt-Ltd.), and KOH (Merck).

### 2.2. Synthesis of MWCNT-supported Fe and FeCo electrocatalysts

All the electrocatalysts, C/Fe, C/Co, C/Fe_50_Co_50_, C/Fe_84_Co_16_, C/Fe_91_Co_9_, and unsupported Fe_50_Co_50_ were synthesized by the impregnation reduction method. MWCNT powder in required amount was added to the 200 ml milli-Q water and ultrasonicated for 15 min using a Digital Ultrasonicator Cleaner (Model No.: LMUC-3, Frequency: 40KHz, Ultrasonic power:100W). The metal precursors, Fe(NO_3_)_3_.9H_2_O and Co(NO_3_)_2_.6H_2_O were then added to the respective carbon solutions and stirred for 1 h. Appropriate quantity of 0.05 M NaBH_4_ (Merck) solution was added, drop by drop, to the mixture with vigorous stirring for the complete reduction of metals from the precursor salt. Then the solution was filtered and cleaned. Finally, it was dried overnight in a vacuum oven for 80°C.

### 2.3. Physical characterization

X-ray diffraction (XRD) was acquired by a BRUKER AXS, Model D8 by help of Cu Ka radiation (λ = 1.5405 Å) and scanned with 2θ (10° to 90°) values with a 2θ step size of 0.02°. The surface morphology was studied by JSM- 7610F Field Emission Scanning Electron Microscope. FTIR analysis was conducted with the help of a PerkinElmer FT-IR/FIR Spectrophotometer. A confocal Raman microscope (T64000, J-Y Horiba) was used to obtain Raman Spectra with the DPSS Laser (Diode Pump Solid State) having Excitation wavelength: 532nm.

### 2.4. Electrochemical studies

The electrode fabrication process commenced with an electrocatalyst ink through a mixture of the appropriate electrocatalysts and ethanol (Merck); after that, the mixture was sonicated for approximately 3 min to make a mixture of slurry. This slurry mixture was fabricated onto the carbon block (surface area 0.16 cm^2^). The loading of the catalyst is maintained at 3.47 mg/cm^2^. After that, using those fabricated carbon blocks as the working electrode, Pt wire as the counter electrode, and Ag/AgCl as the reference electrode in a three-electrode assembly, electrochemical analyses such as Cyclic Voltammetry (CV), Linear Sweep Voltammetry (LSV), Chronoamperometry (CA), and Electrochemical impedance spectroscopy (EIS) were performed. These analyses were performed by a potentiostat, EmStat4 (PalmSens BV, The Netherlands), with the PS Trace 5.9 software. Throughout these experiments, 1 M of Ethanol and 0.5 M of KOH were used as electrolyte, CV and LSV were employed in the −0.1 V to +0.5 V potential range. Chronoamperometry was conducted at −0.1 V for 3600 s. EIS employed 43 data points that ranged in frequency between 100 mHz to 80 kHz. The amplitude of the a.c. signal measured 9 mV.

## 3. Results and discussion

### 3.1. Physical characterization

#### 3.1.1. X-ray diffraction analysis

**Figure 1:**
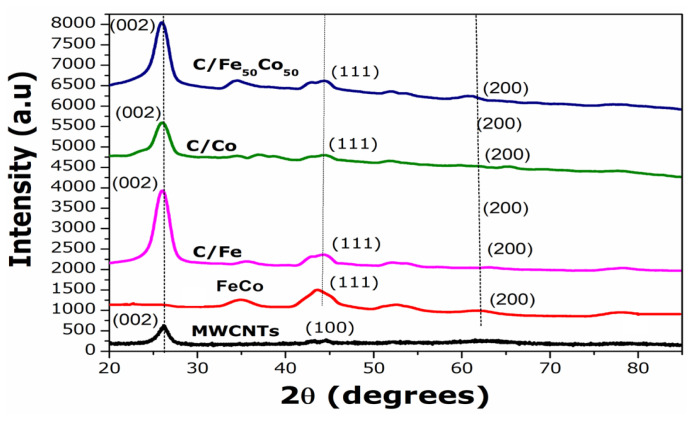
X-ray diffraction patterns of Carbon (MWCNTs), Fe_50_Co_50_, C/Fe, C/Co, and C/Fe_50_Co_50_ electrocatalysts.

Figure 1 shows the XRD patterns of MWCNTs, Fe_50_Co_50_, C/Fe, C/Co, and C/Fe_50_Co_50_ electrocatalyst. Fe_50_Co_50_, C/Fe, C/Co, and C/Fe_50_Co_50_ exhibit the characteristic peaks for the face-centered cubic (fcc) crystal structure of Fe and Co. MWCNTs exhibited diffraction peaks at 26.23°, 43.20° corresponding to the (002) and (100) planes. For C/Fe diffraction peaks appear for corresponding planes (111), (200), and (220) at 44.32°, 53.80°, and 78.34° respectively. For monometallic Cobalt catalyst, peaks are shown at 44.53°, 51.91°, and 78.00°. It is evident that after incorporation of Co to Fe surface, 2θ values are shifted to higher range for C/Fe_50_Co_50_ catalyst as 2θ values appear at 44.56°, 52.09°, and 77.55° for (111), (200), and (220) plan respectively. There is no peak exhibited for hcp or fcc structure of Co. Using Debye-Scherrer formula crystalline size is calculated in the nanometer range. All the parameters indicate the addition of Co to the Fe lattice desirable for FeCo alloy formation in nanometer ranges, and the proper incorporation of Co in the Fe crystal matrix, which is beneficial for Ethanol Oxidation Reaction.

#### 3.1.2. Scanning electron microscopy (SEM)

**Figure 2:**
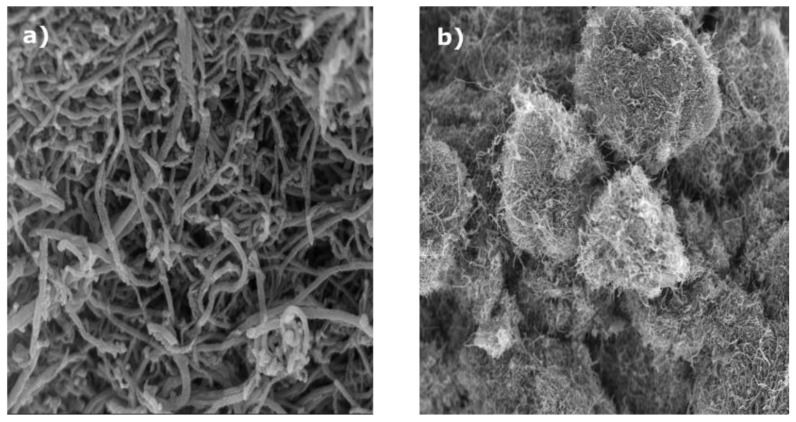
(a, b) Scanning electron microscopy (SEM) images of C/Fe_50_Co_50_ and C/Fe.

Through SEM study, surface morphology of the chemically synthesized catalyst is investigated. In Figure 2a), C/Fe_50_Co_50_ owns small edges nano ribbons with micrometers of length on the surface, those are self-constructed by FeCo metal alloy. Figure 2b) shows that the C/Fe possesses agglomeration rough and bright metallic globules on the catalyst surface.

#### 3.1.3. Fourier transform infrared (FTIR) spectroscopy

**Figure 3:**
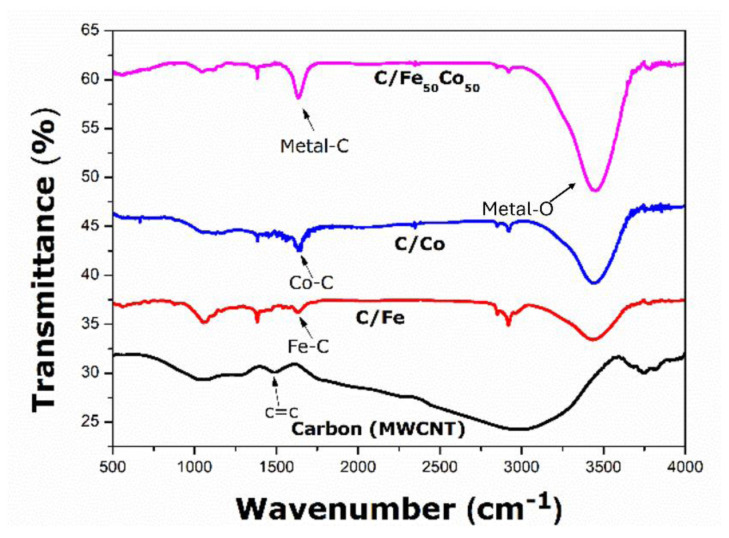
FTIR spectra for Carbon (MWCNTs), C/Fe, C/Co and C/Fe_50_Co_50_ electrocatalysts.

FTIR assists in identifying the bonds formation in the synthesized catalysts from their transmittance peaks. Figure 3 demonstrates the FTIR plots of Fe_50_Co_50_, MWCNTs, C/Fe_50_Co_50_, C/Co, and C/Fe catalysts. Peaks in pure MWCNTs correspond to C=C stretching at around 1510 cm^−1^. Peaks in Figure 3 at around 1570–1600 cm^−1^ illustrate that the C/Co, C/Fe, and C/Fe_50_Co_50_ catalysts may represent metal-carbon interactions. Therefore, alterations in the MWCNTs’ electronic environment as a result of metal deposition may be indicated by a shift in the C=C stretching vibration. The metal alloy oxide bond formation is observed for at ~3443 cm^−1^. It can be observed that there is a change in peak intensities with the inclusion of 50% Co with 50% Fe into the binary catalyst, which causes a shift in peak intensities, that suggests a greater degree of interaction between the metal and the carbon support. A shift in wavenumber to a higher value can indicate a greater electron density surrounding the MWCNTs. Additionally, it suggests that the incorporation of Co with Fe into the catalyst reinforces the oxophilic nature of metal, which is crucial for the ethanol oxidation reaction on the surface of the electrocatalyst [11].

#### 3.1.4. Raman analysis

**Figure 4:**
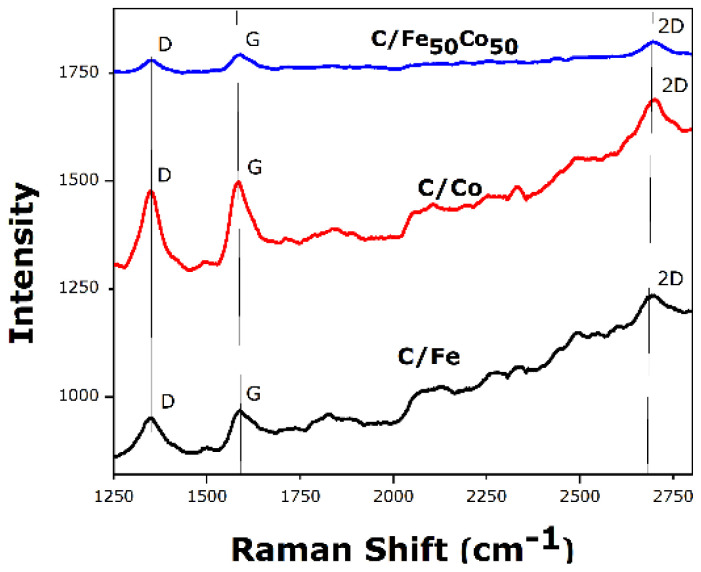
Raman spectra for C/Fe, C/Co and C/Fe_50_Co_50_ electrocatalysts.

Figure 4 reveals the structural change, phase information in catalyst using Raman spectroscopy. Two distinct peaks are observed at around ~1349.90 cm^−1^ (D band) and ~ 1588.75 cm^−1^ (G band) in accordance with the disordered carbon and the hybridized sp^2^ carbon for C/Fe_50_Co_50_ catalyst. Also, a 2D band is observed at 2696 cm^−1^ for binary catalyst. It is evident that D, G band of binary 50:50 catalyst is shifted toward right side from the monometallic Fe and Co. The ratio indicates the domain size of sp^2^ and crystal structure of carbon, which is compared in Table. This suggests that there may be additional nanocomposite structure present in bimetallic C/Fe_50_Co_50_, as seen by the Fe, Co nanoparticles that directly interact with the sp^2^ hybridized structure of the MWCNT and interfere with its inherent graphitic structure [12–13].

### 3.2. Electrochemical studies

#### 3.2.1. Cyclic voltammetry

**Figure 5:**
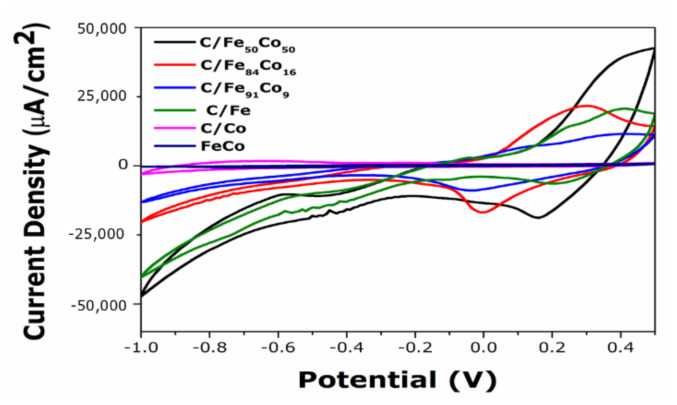
Cyclic voltammograms of C/Fe, C/Co, C/Fe_50_Co_50_, C/Fe_84_Co_16_, C/Fe_91_Co_9_ and Fe_50_Co_50_ in 0.5 M KOH and 1M of EtOH recorded at scan rate 0.1 V/s.

Figure 5 demonstrates the Cyclic Voltammograms of C/Fe, C/Co, unsupported Fe_50_Co_50_, and C/FeCo bimetallic alloy catalysts in different Fe and Co ratios in 0.5 M KOH and 1 M ethanol solutions at 0.1 V/s scan rate. A well-defined oxidation peak is exhibited during the oxidation of ethanol in Figure 2. There is no oxidation that occurs below −0.40 V due to electrode surface blocking by carbonaceous materials, intermediate get oxidized after this potential, and anodic peak exhibited. It is observed that onset potential is shifted towards the left side for C/Fe_50_Co_50_ at −0.43 V. For C/Fe_84_Co_16_ and C/Fe_91_Co_9_, the onset potential is 0.02 V and −0.07 V, respectively, which may be explained by the fact that the increase in Co% into binary MWCNTs supported catalysts with higher catalytic activity. It is demonstrated that the binary catalyst exhibits a reduced catalytic activity when 9% Co and 91% Fe are added into MWCNTs. This is because the lower percentage of Co inhibits the accumulation of oxygen species on the catalyst surface, resulting in diminishing the selectivity of EOR. For unsupported Fe_50_Co_50_catalyst, it shows very low current density with very high onset potential. C/Fe_50_Co_50_ indicates better catalytic activity as it has the lowest onset potential than other catalysts. It denotes that binary C/Fe_50_Co_50_ catalyst is capable of adsorbing water and dissociating to form OH species at lower anodic potential than unsupported Fe_50_Co_50_ alloy, monometallic Fe and Co as result ethanolic species can be oxidized at low potential. Addition of optimum Co to Fe surface is beneficial as formation of [−O–Co–O–Co–O–] on the surface promotes the ethanol oxidation and tolerance of CO poison. The disparity between the peak current [(i_p_, _f_), (i_p_, _r_)] observed during the forward reaction and the backward reaction, suggests irreversible electron transfer reactions in both directions [14–18].

#### 3.2.2. Linear sweep voltammetry analysis

**Figure 6:**
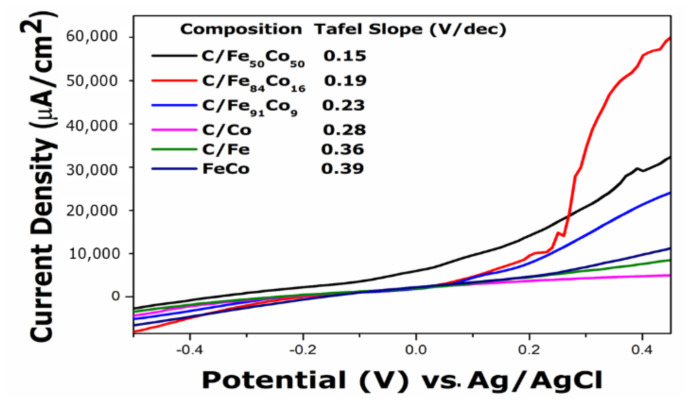
Linear Sweep voltammograms of C/Fe, C/Co, Fe_50_Co_50_, C/Fe_50_Co_50_, C/Fe_84_Co_16_, and C/Fe_91_Co_9_ in 0.5 M KOH and 1M of EtOH recorded at scan rate 0.1 V/s.

A comparative analysis of the linear sweep voltammograms (LSV) of the C/Fe, C/Co, Fe_50_Co_50_, and C/FeCo electrocatalysts with all ratios highlights the significant influence in ethanol oxidation. From Figure 6, potentiodynamic polarization behaviors for ethanol oxidation are explained. Tafel slopes obtained from potentiodynamic polarization studies at 0.1 V/sec scan rate show that portraited the C/Fe_50_Co_50_ is exhibiting higher electrocatalytic activity toward ethanol oxidation. It is evidenced that unsupported Fe_50_Co_50_ has the highest Tafel slope value, which is related to the slower oxidation kinetics. The lowest Tafel slope value of C/Fe_50_Co_50_ catalyst amongst all catalysts shows best polarization current densities also lead to lower overpotential because of the low energy required to achieve for electrochemical process and the synergistic effect of Fe and Co enhances the electro-oxidation of ethanol in an alkaline environment [19].

#### 3.2.3. Chronoamperometric Analysis

**Figure 7:**
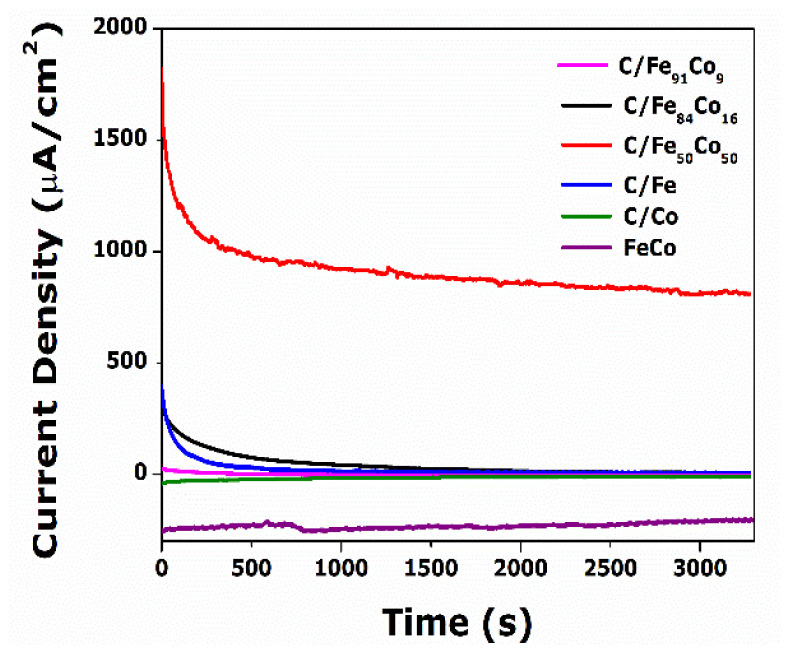
Chronoamperograms of C/Fe, C/Co, Fe_50_Co_50_, C/Fe_50_Co_50_, C/Fe_84_Co_16_ and C/Fe_91_Co_9_ recorded at −0.1 V.

It is evident from the Cyclic Voltametric research and the Linear Sweep Voltametric study that Ethanol oxidation produces intermediates that are adsorbed on toxic catalyst surfaces. They thereby lose stability and experience a slowdown in catalytic activity. Potential controlled chronoamperometric methods were employed to examine surface deactivation. Figure 7 illustrates the current density-time reciprocation of various monometallic, unsupported FeCo, and bimetallic carbon-supported Fe-based Co alloy catalyst of different compositions for EOR. The study was conducted at a fixed potential of −0.1 V. The resultant curve shows that the current drops before reaching a steady state. Because of the electron transfer caused by the catalyst’s stepped potential, a faradaic current is seen. The high charged current generated gradually decreases exponentially with time. Due to the less dispersed, highly agglomerated FeCo metal alloys in the unsupported Fe_50_Co_50_ catalyst and due to chemisorbed carbonaceous species during alcohol oxidation, unsupported catalyst shows very low current density. This indicates the MWCNTs support is crucial for EOR because it has a larger surface area, which can assist in enhancing the reaction kinetics through the metal-support interaction. Among the other catalysts, carbon supported by 50:50 FeCo catalyst ratio exhibits the greatest stability. This study implies that for Fe to act as an electrocatalyst via oxygen transfer, a sufficient number of active Fe sites for CO adsorption is essential [20–21].

#### 3.2.4. Electrochemical impedance spectroscopy (EIS)

**Figure 8:**
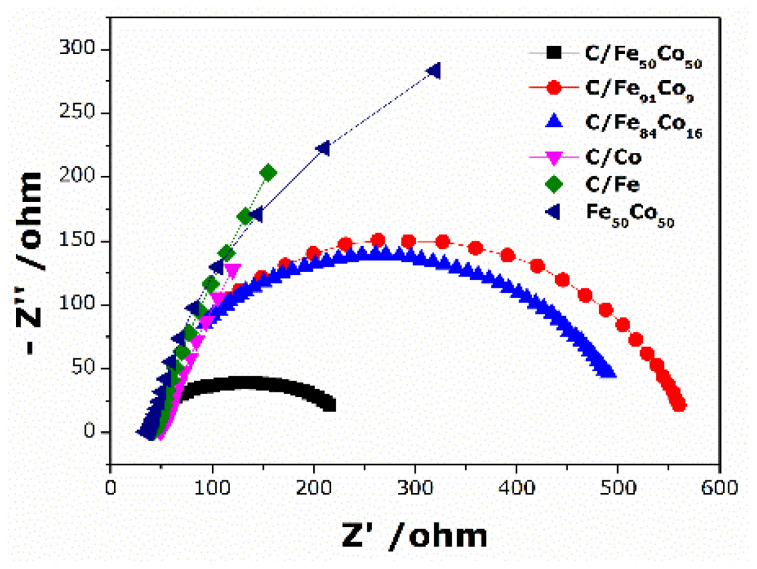
Nyquist plots of ethanol oxidation at a potential of −0.1 V.

Nyquist plots depicting the ethanol oxidation process in 1 M ethanol and 0.5 M KOH on C/Fe, C/Co, unsupported Fe_50_Co_50_, and different ratio of C/FeCo electrodes supported by MWCNT at −0.1 V vs Ag/AgCl are shown in Figure 8. The emergence of an arc in the complex-plane plots of all the electrocatalysts suggests the presence of a resistive component. The width of the semicircle in the diagram, which indicates the charge transfer resistance (R_ct_), is correlated with the kinetics of the charge transfer process [22]. In Figure 8, the charge transfer resistance trend is displayed in the following order: C/Co> C/Fe> unsupported Fe_50_Co_50_> C/Fe_84_Co_16_ > C/Fe_91_Co_9_ > C/Fe_50_Co_50_. Co is added to the carbon supported Fe surface, increasing the charge transfer kinetics. The C/Co and C/Fe electrodes may exhibit a decrease in the current density due to a decrease in the number of active sites on the surface caused by the occurrence of CO intermediates, which may be observed. Due to the strong formation of chemisorbed hydroxy species, C/Fe_50_Co_50_ has the lowest R_ct_, indicating that there is less inhibiting by intermediate species. Therefore, a high electrochemically active area is provided by the optimal Co concentration in C/Fe_50_Co_50_, which presumably accelerates forward the oxidation process.

### 3.3. Mechanism

The mechanism of electro-oxidation of ethanol on MWCNT supported bimetallic FeCo surface is proposed where MWCNT boosts the electrocatalytic activity as it possesses high electrical conductivity, a substantial surface area, and sufficient stability under operational conditions [23,24]. Ethanol oxidation in alkaline environments can proceed through multiple concurrent pathways, resulting in the formation of acetate (through steps 4, 5, 6, 7, and 8), [25] acetaldehyde (via steps 9, 10, and 11), [26] or CO_2_ (step 12). However, it’s worth noting that the last pathway involves the interaction of a deactivating species, CO_ad_, with OH_ad_/OH^−^ species, which, in alkaline conditions, leads to the generation of carbonate [26]. Furthermore, it’s important to acknowledge that the steps responsible for the cleavage of C–C bonds to produce CO_2_ in alkaline environments are not well-documented in the existing literature.[Fig f9-tjc-49-01-45]

Nonetheless, it has been suggested that acetaldehyde serves as a pivotal intermediate in the formation of acetic acid, which then exists as an acetate anion within alkaline conditions (through steps 13 and 14).[Fig f10-tjc-49-01-45]

## 4. Conclusion

According to the comparative analysis of these electrocatalysts, the synthesized bimetallic catalysts produced using the impregnation reduction method are both catalytically and electrochemically active. The nano crystallite size obtained by using Debye Scherrer’s equation results in increase in catalytic area; and C/Fe_50_Co_50_ exhibits the best electrocatalytic activity toward the oxidation of both ethanol compared to the other catalysts because it has the lowest onset potential and the highest peak current density for ethanol oxidation, as determined by cyclic voltammogram analysis. The catalyst C/Fe_50_Co_50_ had the greatest peak current density, indicating a higher degree of ethanol oxidation confirmed by both structural, morphological, and electrochemical studies.

## Figures and Tables

**Scheme 1 f9-tjc-49-01-45:**
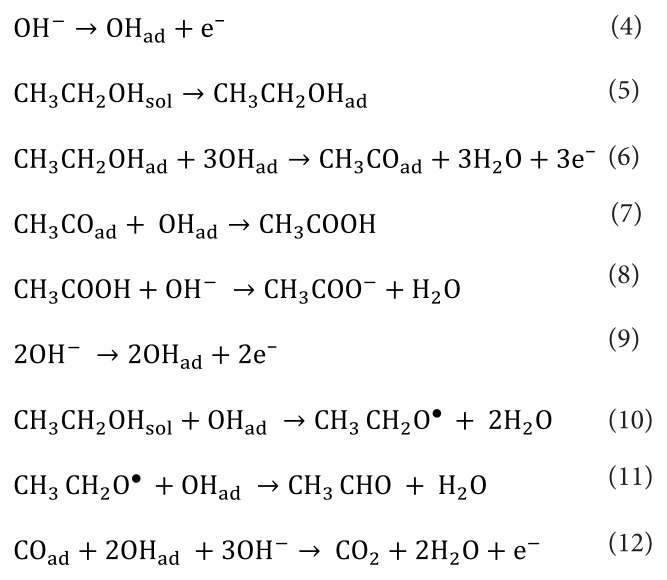
Proposed mechanism of electro-oxidation of ethanol on bimetallic FeCo surface under alkaline condition. Species with the subscript ads implies adsorbed species on bimetallic FeCo surface.

**Scheme 2 f10-tjc-49-01-45:**
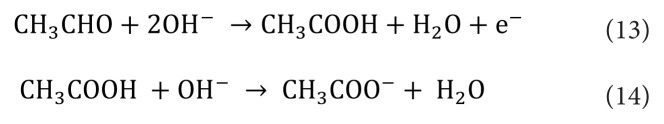
Mechanism of electro-oxidation of acetaldehyde to acetic acid on bimetallic FeCo surface under alkaline condition.

**Table:** Ratio of intensity of D band and G band.

Composition	I_D_/I_G_
C/Fe_50_Co_50_	0.94
C/Co	0.93
C/Fe	0.97
